# Construction of ceRNA regulatory networks for active pulmonary tuberculosis

**DOI:** 10.1038/s41598-024-61451-2

**Published:** 2024-05-08

**Authors:** Qifeng Li, Tao Xin, Zhigang Liu, Quan Wang, Lanhong Ma

**Affiliations:** 1 Xinjiang Institute of Pediatrics, Children’s Hospital of Xinjiang Uygur Autonomous Region, NO. 393, Aletai Road, Shayibake District, Urumqi, 830054 Xinjiang China; 2 Department of Pediatrics, The Eighth Affiliated Hospital of Xinjiang Medical University, Urumqi, 830049 China; 3 Department of Thoracic Surgery, The Eighth Affiliated Hospital of Xinjiang Medical University, Urumqi, 830049 China; 4 Department of Clinical Laboratory, The Eighth Affiliated Hospital of Xinjiang Medical University, Urumqi, 830049 China; 5 Department of Pediatrics, Children’s Hospital of Xinjiang Uygur Autonomous Region, Urumqi, 830054 China

**Keywords:** Pulmonary tuberculosis, Transcriptome sequencing, ceRNA network, Helper T cell differentiation, Biocatalysis, Respiratory tract diseases

## Abstract

Delayed diagnosis in patients with pulmonary tuberculosis (PTB) often leads to serious public health problems. High throughput sequencing was used to determine the expression levels of lncRNAs, mRNAs, and miRNAs in the lesions and adjacent health lung tissues of patients with PTB. Their differential expression profiles between the two groups were compared, and 146 DElncRs, 447 DEmRs, and 29 DEmiRs were obtained between lesions and adjacent health tissues in patients with PTB. Enrichment analysis for mRNAs showed that they were mainly involved in Th1, Th2, and Th17 cell differentiation. The lncRNAs, mRNAs with target relationship with miRNAs were predicted respectively, and correlation analysis was performed. The ceRNA regulatory network was obtained by comparing with the differentially expressed transcripts (DElncRs, DEmRs, DEmiRs), then 2 lncRNAs mediated ceRNA networks were established. The expression of genes within the network was verified by quantitative real-time PCR (qRT-PCR). Flow cytometric analysis revealed that the proportion of Th1 cells and Th17 cells was lower in PTB than in controls, while the proportion of Th2 cells increased. Our results provide rich transcriptome data for a deeper investigation of PTB. The ceRNA regulatory network we obtained may be instructive for the diagnosis and treatment of PTB.

## Introduction

Pulmonary tuberculosis (PTB) is an infectious disease caused by invasion of lung tissue by *Mycobacterium tuberculosis*^1^. PTB remains a formidable challenge to global health, being the deadliest infectious disease despite advances in vaccination, therapy, and diagnostics^2^. As the second-largest burden of PTB and multidrug resistance, China faces significant challenges in tackling this disease^3^. PTB accounts for more deaths worldwide than any other infectious disease, and is essentially curable if detected early and treated effectively^4^. While etiological testing is the standard for diagnosing PTB, it is hampered by several limitations^5^.

Recent studies have shed light on the role of immune responses and inflammatory factors in the progression of PTB, opening avenues for potential diagnostic and therapeutic targets^6,7^. PTB is known to impair immune function and cause systemic wasting^8,9^, with excessive inflammatory responses exacerbating its severity and complicating treatment^10,11^.

In the realm of molecular biology, gene regulation plays a crucial role in maintaining cellular function and has been implicated in the pathogenesis of complex diseases like PTB^12^. Noncoding RNAs (ncRNAs), particularly long noncoding RNAs (lncRNAs) and microRNAs (miRNAs), have emerged as significant players in the immune response to PTB^13^. LncRNAs, for instance, are key regulators in the inflammatory response to infection, particularly influencing T cell responses^14^, while miRNAs have been increasingly recognized for modulating host-TB bacilli interactions through immune pathways^15^. Competing endogenous RNAs (ceRNAs) offer a novel mechanism for regulating miRNA activity, acting as sponges through their miRNA response elements^16^. These findings suggest the potential of the ceRNA network as a biomarker for PTB diagnosis and treatment.

However, despite these advances, the specific roles and interactions of ncRNAs, lncRNAs, and ceRNAs in PTB remain underexplored. Previous studies have laid a foundation, but gaps persist in understanding the full scope of their regulatory functions and potential as diagnostic and therapeutic targets^17^. This study aims to fill these gaps by focusing on the ceRNA network in PTB. Through whole transcriptome sequencing of patients with active PTB, we seek to identify lncRNA-associated ceRNA regulatory networks, potentially unveiling novel biomarkers for PTB. Such insights could improve the early diagnosis and treatment of this global health threat.

## Materials and methods

### Participants

A total of 37 patients with active PTB and 37 healthy controls were recruited from Children’s Hospital of Xinjiang Uygur Autonomous Region (Table 1). Among this, 7 cases underwent biopsies due to acute hemoptysis with non-prominent clinical symptoms. The biopsy needle extracted a small amount of tissue from the patient’s lungs for testing. Additionally, lesions refer to areas of granulomatous disease as identified pathologically, and adherent refers to non-granulomatous areas, specifically the tissue surrounding the granulomas (3 cm away from the granulomatous regions). Three patients were randomly selected, including both their lesions and adjacent lung tissues. Peripheral blood samples were obtained from 37 patients with active PTB and 37 healthy controls.

**Table 1:** Baseline characteristics of participants.

		Healthy controls (n = 37)	Active pulmonary tuberculosis (n = 37)	*t*	*P* value
Age, years		32.50 ± 11.15	31.50 ± 10.56	0.42	0.68
Sex					
	Male	19 (51.4%)	19 (51.4%)	0.00	1.00
	Female	18 (48.6%)	18 (48.6%)		
CD4 count, cells per μL		384.86 ± 124.04	315.21 ± 99.63	2.34	0.03
Haemoglobin concentration, g/dL					
	Male	14.25 ± 1.38	11.29 ± 1.12	6.28	< 0.01
	Female	12.28 ± 1.10	10.21 ± 1.18	9.04	< 0.01
Leucocyte count, × 10^9^ cells per L		6.91 ± 2.42	9.75 ± 2.88	− 4.48	< 0.01
BMI, kg/m^2^		20.05 ± 4.26	19.81 ± 4.38	0.21	0.84
Previous tuberculosis				25.71	< 0.01
	No	30 (100%)	12 (40%)		
	Yes	0	18 (60%)		

*BMI* body mass index.

Human studies were reviewed and approved by the Ethics committee of Children’s Hospital of Xinjiang Uygur Autonomous Region (No. 2019030622), and adhered to the Declaration of Helsinki. Written informed consent was obtained from all patients.

Inclusion Criteria for patients with PTB: Confirmed diagnosis of pulmonary tuberculosis based on clinical, radiological, and microbiological evidence. Exclusion Criteria for patients with PTB: Co-infection with HIV or other immunocompromising conditions; History of anti-tuberculosis treatment in the past six months; Presence of other chronic pulmonary diseases. Selection Criteria for Healthy Controls: No history or clinical signs of tuberculosis or other chronic illnesses; Age and sex-matched to the patients with PTB group.

### RNA preparation

Total RNA was extracted from lesions and adjacent healthy lung tissues using Trizol (Invitrogen, MA, USA). RNA concentration was quantified using Nanodrop. The integrity and quantity of the RNA were assessed using an Agilent 2100 Bioanalyzer (Agilent Technologies, CA, USA) and a NanoDrop (Thermo, MA, USA), respectively, ensuring a RNA Integrity Number (RIN) > 7.0 for downstream applications.

### Transcriptome sequencing

Sequencing libraries were prepared from the high-quality RNA samples using the NEBNext^®^ Ultra^™^ RNA Library Prep Kit for Illumina^®^ (New England Biolabs, USA), following the manufacturer’s instructions. Briefly, mRNA was enriched and fragmented, followed by first-strand cDNA synthesis using reverse transcriptase and random hexamer primers. Second-strand cDNA synthesis incorporated dUTP to mark the strand used for sequencing. End repair, A-tailing, adapter ligation, and index addition were performed before PCR enrichment of the final library. Libraries were validated for quality and quantified using the Agilent Bioanalyzer 2100 and qPCR. Size selection was targeted to cDNA fragments of 250–300 bp. Following library preparation, the sequencing was performed on an Illumina PE150 platform using the S4 flow cell and Illumina’s standard sequencing chemistry. This targeting a sequencing depth of approximately 30 million reads per sample.

For small RNA sequencing, libraries were generated using NEBNext^®^ Multiplex Small RNA Library Prep Set for Illumina^®^ (NEB, USA). DNA fragments corresponding to 140–160 bp, representing small RNA ligated to adaptors, were recovered and purified. The quality of these libraries was also assessed using the Agilent Bioanalyzer 2100 system. The libraries were sequenced on the same Illumina PE150 platform, targeting a sequencing depth of around 10 million reads per sample to sufficiently capture the small RNA profile.

### Data processing

Raw reads of fastq format were firstly processed through in-house perl scripts. Then, a certain range of length from clean reads with high quality was chosen to do downstream analyses. Reference genome and gene model annotation files were downloaded from genome website directly (https://ftp.ensembl.org/pub/release-97/fasta/homo_sapiens/dna/). Index of the reference genome was built using Hisat2 (v2.0.5)^18^. On the other hand, the small RNA tags were mapped to reference sequence^19^ without mismatch to analyze their expression and distribution on the reference.

### Quantification of transcript expression levels and differential analysis

The reads numbers mapped to each transcript were counted using feature Counts v1.5.0-p3. Then FPKM (Fragments Per Kilobase of transcript sequence per Millions) of each transcript was calculated based on the length of the transcript and reads count mapped to this transcript. The miRNA expression levels were estimated by TPM (transcript per million). Principal component analysis (PCA) was performed using prcomp R function.

Differential expression analysis between lesions and adjacent lung tissues was performed using the DESeq R package^20^ for miRNAs, lncRNAs and mRNAs. Benjamini–Hochberg method was used to select false discovery rate (FDR) < 0.05 to assign as differentially expressed lncRNAs (DElncRs) and differentially expressed mRNAs (DEmRs). A *P* < 0.05 were assigned as differentially expressed miRNAs (DEmiRs).

### Construction of the ceRNA network

The lncRNAs with target relationship with expressed miRNAs in all samples were predicted according to miRanda software (v22)^21^. The mRNAs predicted by miRanda and RNAhybrid softwares (v2.1.2)^22^ to have a target relationship with the expressed miRNAs in all samples and identified the intersection mRNAs. Further, the Pearson’s correlation coefficient was calculated for all lncRNAs or mRNAs that had a targeting relationship with miRNAs, the threshold was set as negative correlation. MiRNAs, mRNAs with a targeted relationship and negatively correlated expression with this miRNA, and lncRNAs with a targeted relationship and negatively correlated expression with this miRNA were screened, respectively. Finally, we constructed lncRNA-miRNA-mRNA ceRNA networks.

### Functional annotation analysis

Gene Ontology (GO) analysis was performed using clusterProfiler R package^23^ for DEmRs. Kyoto Encyclopedia of Genes and Genomes (KEGG) pathways^24^ were enriched by the Enrichr R package^25^. We used *P* < 0.05 to test the significant enrichment results.

### Quantitative real-time PCR

Reverse transcription of total RNA extracted from 10 lesions and adjacent healthy lung tissues into cDNA was performed using First-Strand cDNA Synthesis SuperMix (Takara, Dalian, China). Quantitative real-time PCR (qRT-PCR) then was performed with specific primers using SYBR Green PCR kit (Invitrogen), according to the manufacturer’s protocol. Expression of GAPDH was used as internal standard for lncRNAs and mRNAs, U6 was used as internal standard for miRNAs. Results were analyzed using 2^−ΔΔCt^ method. Primer sequences which designed by sagan corporation are shown in Supplemental Table 1. A *P* < 0.05 was considered significant difference.

### Flow cytometry

The whole blood samples of 30 patients with PTB and 30 controls were collected. Cell surface markers were then stained with antibodies against CD4 FITC (Cat 555346), CD196 PE (Cat 559562), CD183 ECD (Cat 551128) from BD (San Diego, USA), according to standard procedures. The gating strategy was CD4 + CD183 + CD196- for Th1, CD4 + CD183− CD196 for Th2, and CD4 + CD183-CD196 + for Th17^26^. The cells were treated with red blood cell lysate (BD, California, USA) and washed with PBS twice. Flow cytometry of cells was performed with a BD LSRII flow cytometer and analyzed with FlowJo software (v7).

### Statistical analyses

R (v4.2.3) and Bioconductor (v3.17) were used for bioinformatic analyses. All data are shown as mean ± standard deviation (SD). All data are under normal distribution. Statistical significance between groups was analyzed by Student’s *t*-test using the SPSS software (v19.0). All experiments performed in three times.

### Ethics approval and consent to participate

The study was conducted strictly in accordance with the Declaration of Helsinki and approved by the Ethics Committee of Children’s Hospital of Xinjiang Uygur Autonomous Region (No.2019030622), all respondents under investigation have signed written informed consent.

## Results

### Aberrantly expressed transcripts in PTB

**Figure 1:**
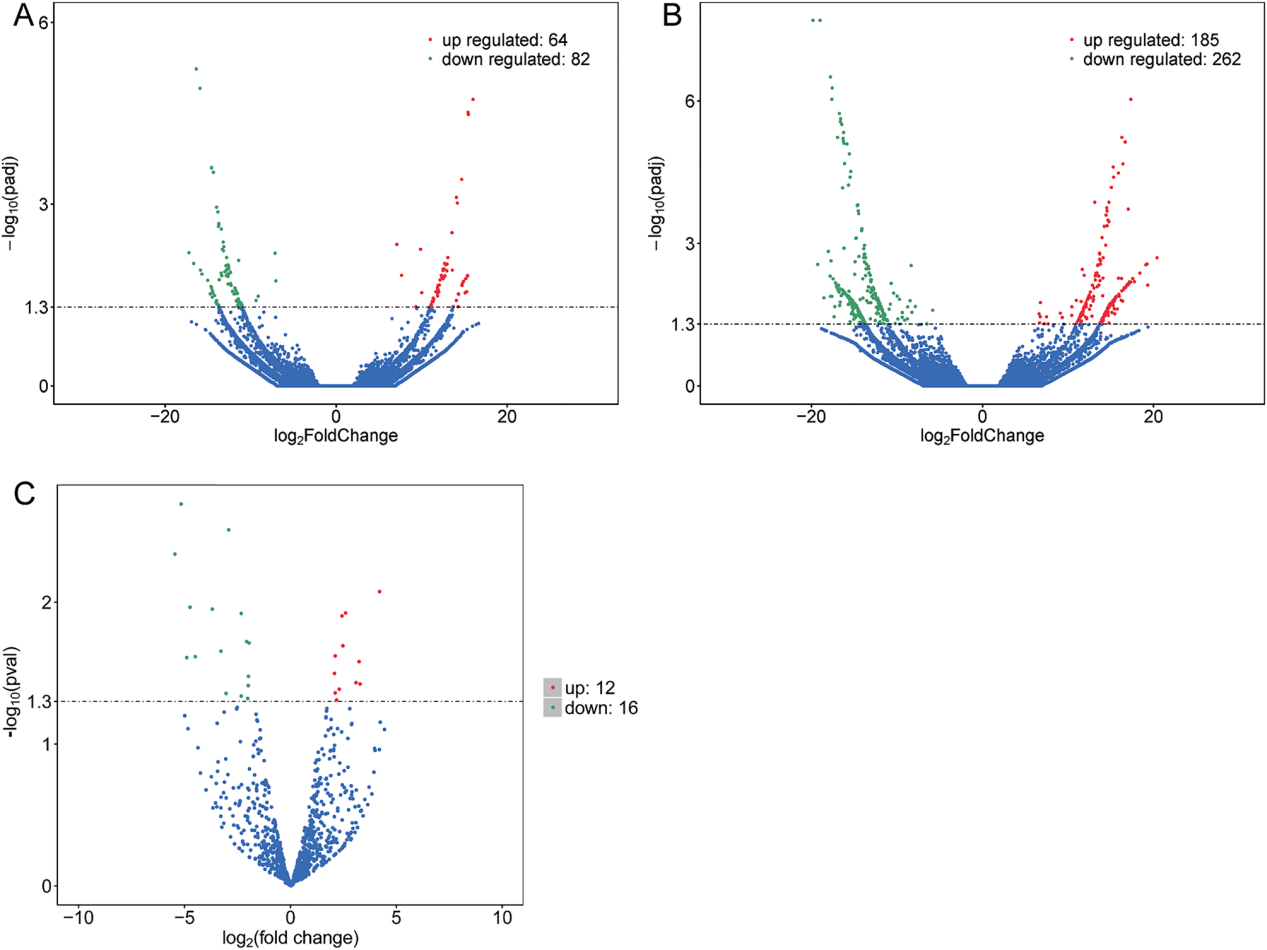
Differentially expressed transcripts between lesions and adjacent healthy lung tissues. (**A**) Volcano plot of differentially expressed lncRNAs. Red dots represent up-regulated expression, green dots represent down-regulated, and blue dots represent non-differential expression. (**B**) Volcano plot of differentially expressed mRNAs. Red dots represent up-regulated expression, green dots represent down-regulated, and blue dots represent non-differential expression. (**C**) Volcano plot of differentially expressed miRNAs. Red dots represent up-regulated expression, green dots represent down-regulated, and blue dots represent non-differential expression.

PCA showed two relatively different distribution patterns between lesions and adjacent healthy groups (Figure S1). To identify aberrantly expressed transcripts in PTB, we performed differential analysis of transcripts expression in lesions and adjacent health tissues. By threshold screening, we identified 146 differentially expressed lncRNAs, including 64 upregulated and 82 downregulated (Fig. 1A). A total of 447 differentially expressed mRNAs were identified, including 185 upregulated and 262 downregulated (Fig. 1B). And 29 differentially expressed miRNAs were also identified, including 12 upregulated and 17 downregulated (Fig. 1C).

### CeRNA networks for pulmonary tuberculosis

**Figure 2:**
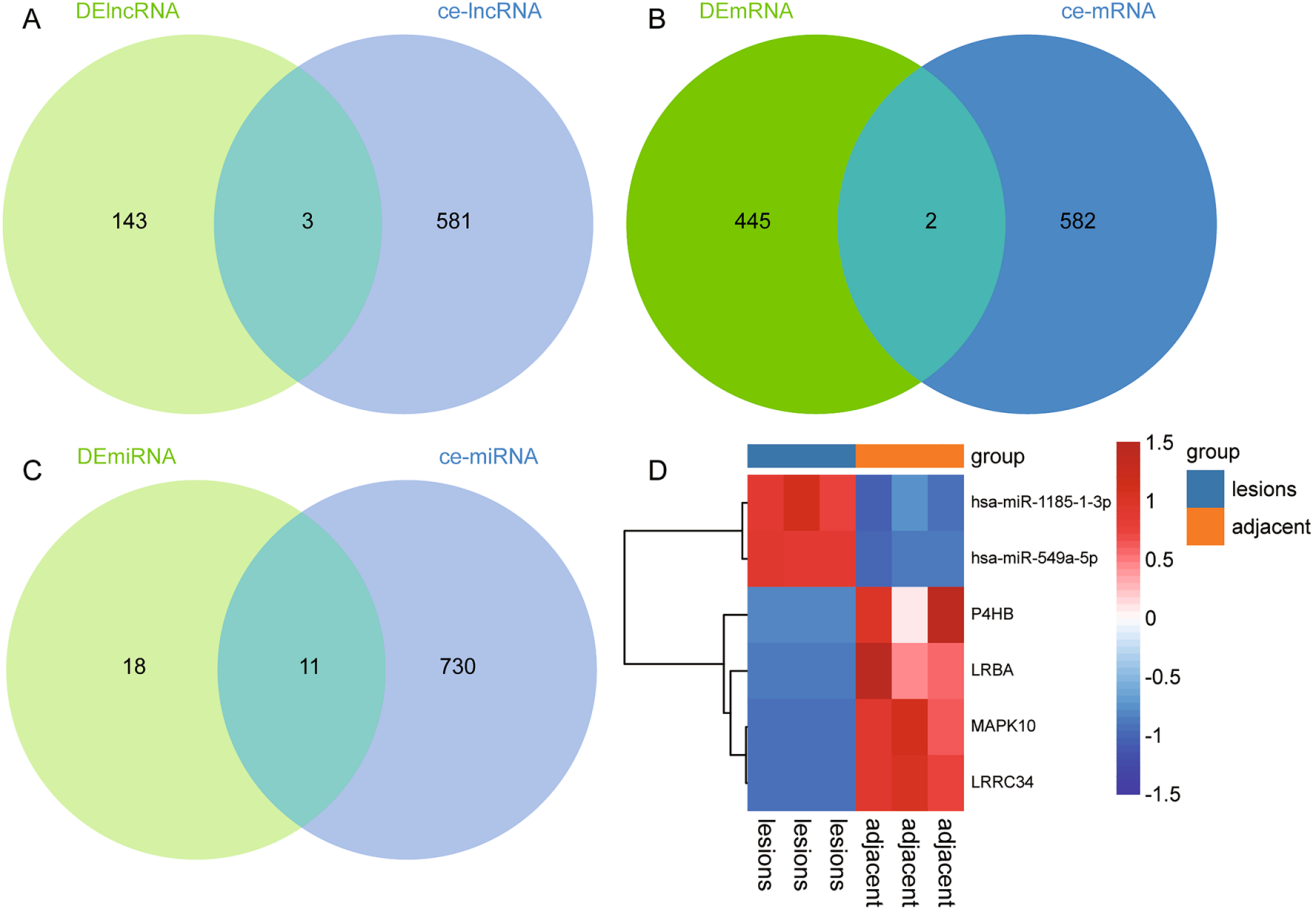
Identification of the ceRNA regulator network in pulmonary tuberculosis. (**A**) Intersection between lncRNAs in ceRNA network and differentially expressed lncRNAs. (**B**) Intersection between mRNAs in ceRNA network and differentially expressed mRNAs. (**C**) Intersection between miRNAs in ceRNA network and differentially expressed miRNAs. (**D**) Heatmap of DElncRs, DEmRs, and DEmiRs of ceRNA regulator network.

Using the online prediction website, we identified lncRNAs and mRNAs that had targeting relationships with miRNAs. Through correlation analysis, we screened the lncRNAs and mRNAs with negative correlation with miRNAs, respectively. Therefore, we constructed lncRNA-miRNA-mRNA networks. Afterward, to identify ceRNA networks that may have regulatory roles in PTB, we compared the transcripts in constructed lncRNA-miRNA-mRNA networks with the differentially expressed transcripts. Further 3 DElncRs (Fig. 2A), 2 DEmRs (Fig. 2B), 11 DEmiRs (Fig. 2C) were identified in the ceRNA networks. Therefore, we constructed two ceRNA regulator networks (LRRC34/hsa-miR-1185-1-3p/MAPK10; P4HB/hsa-miR-549a-5p/LRBA). Among them, hsa-miR-1185-1-3p and hsa-miR-549a-5p were up-regulated in PTB, and LRRC34, P4HB, MAPK10, and LRBA were down regulated in PTB (Fig. 2D).

### Enrichment analysis of DEmRs

**Figure 3:**
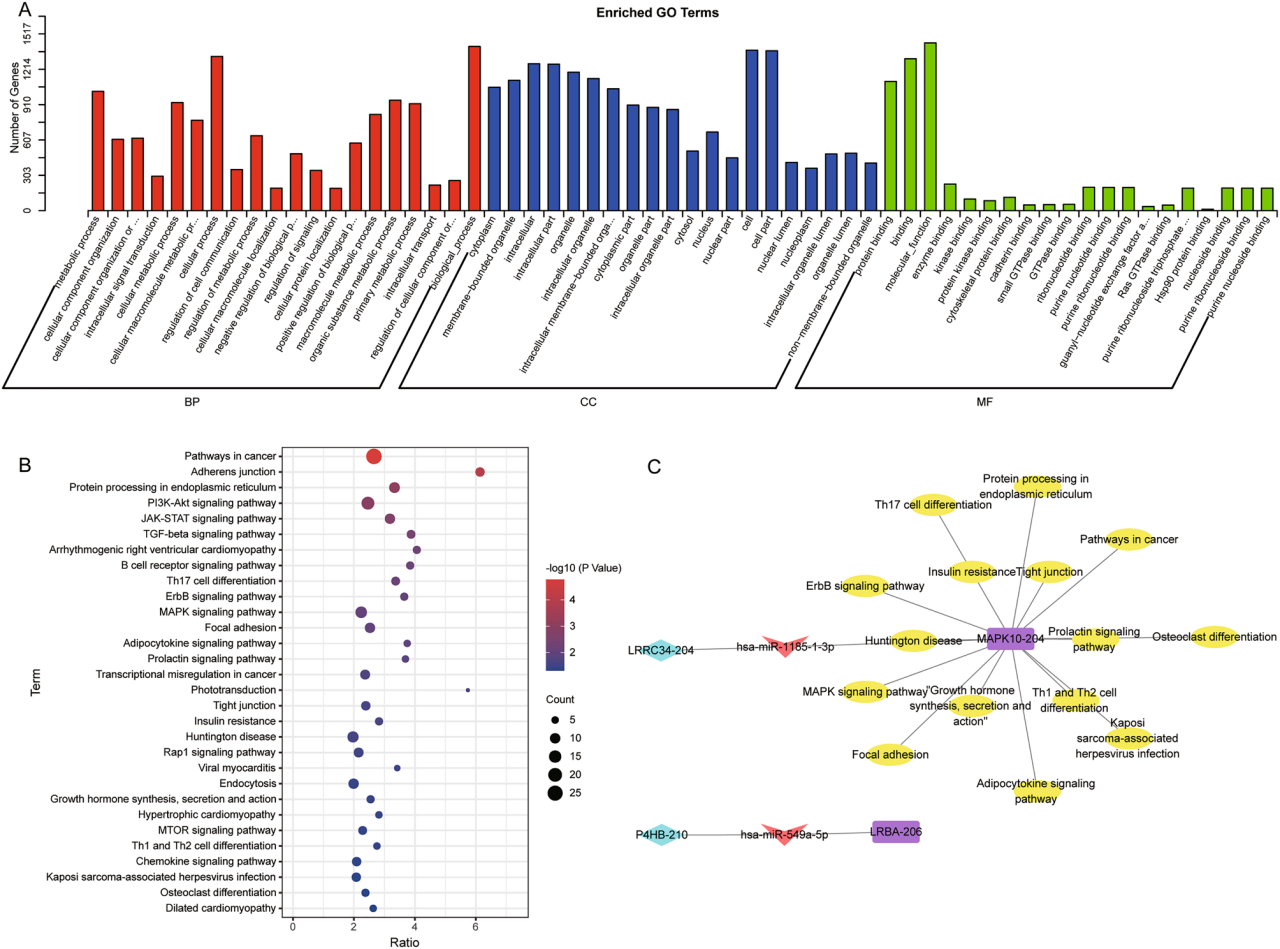
Enrichment of GO and KEGG pathways for DEmRs. (**A**) Classification of top 20 significant GO terms for DEmRs. (**B**) Enrichment of significant KEGG pathways for DEmRs. (**C**) The comprehensive ceRNA regulatory networks in PTB.

To explore the underlying pathological mechanism of PTB, we performed biological function enrichment analysis of DEmRs. GO analysis results (Fig. 3A) showed that metabolic process, cellular component organization, and cellular metabolic process were enriched in biological processes (BP). For cellular component, cytoplasm, membrane-bounded organelle, and intracellular were significantly enriched terms. Major representatives of molecular function included protein binding, binding, and molecular function. In addition, 30 KEGG pathway analysis terms were evaluated, such as adherens junction, PI3K-Akt signaling pathway, JAK-STAT signaling pathway, TGF-beta signaling pathway, B cell receptor signaling pathway, Th1 and Th2 cell differentiation, and Th17 cell differentiation (Fig. 3B, Table S2). Together, we constructed two comprehensive ceRNA regulatory networks (Fig. 3C). As we were more concerned about T cell immunity, LRRC34/hsa-miR-1185-1-3p/MAPK10 contributed to Th1, Th2, and Th17 cell differentiation.

### Experimental validation of significant genes and immune cells

**Figure 4:**
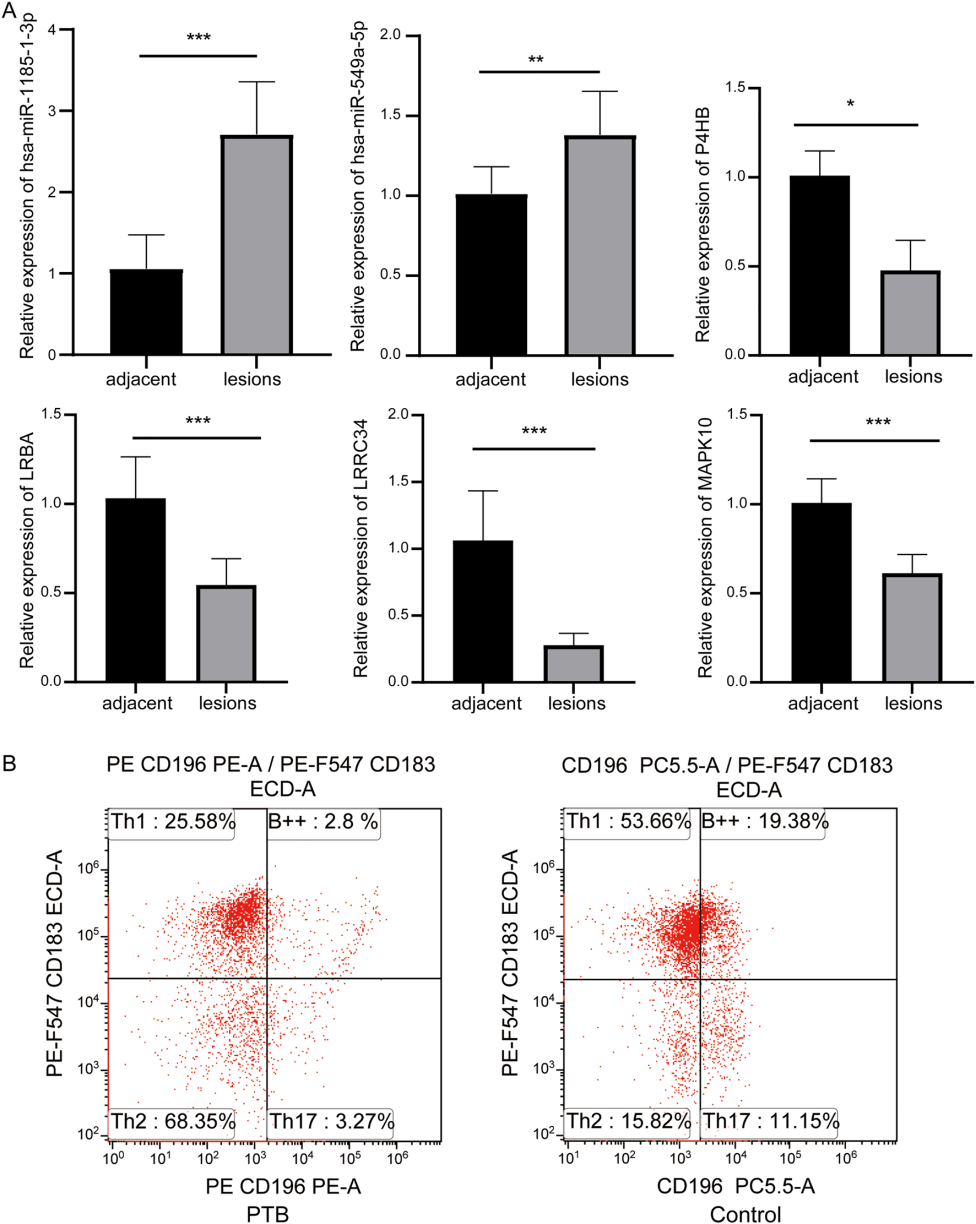
Molecular experiments validate significant analytical results. (**A**) Expression changes of genes in ceRNA regulatory networks in lesions and adjacent healthy lung tissues of patients with PTB through qRT-PCR detection and analyzed by Student’s *t*-test. *P < 0.05, **P < 0.01, ***P < 0.001. (**B**) The proportion change of Th1, Th2, and Th17 cells in patients with PTB and controls detected using flow cytometry.

To further confirm the significant results we obtained, we performed molecular experiments in clinical samples. We examined the expression of genes in the ceRNA regulatory networks in lesions and adjunct health tissues using qRT-PCR experiments (Fig. 4A). Compared with adjacent, hsa-miR-1185-1-3p and hsa-miR-549a-5p showed elevated expression in lesions, and LRRC34, P4HB, MAPK10, and LRBA showed decreased expression in lesions. In addition, we examined the levels of Th1 cells, Th2 cells, and Th17 cells in PTB and healthy controls using flow cytometry (Fig. 4B). Th1 cells and Th17 cells were significantly less abundant in PTB than in controls, while the proportion of Th2 cells was up regulated.

## Discussion

PTB is the leading cause of death worldwide, resulting in increased medical costs and other socioeconomic burdens^27^. Early and accurate diagnosis is of great importance to effectively control and treat PTB infection, and prevent TB from developing into MDR-TB. Understanding the pathogenesis of pulmonary tuberculosis is beneficial for the prevention and treatment of tuberculosis. The discovery of lncRNAs mediated ceRNA regulatory network reveals new insights into PTB related mechanisms and lays a new theoretical foundation for researchers and physicians to develop effective treatments and diagnoses against this highly prevalent disease^28^.

This study used high-throughput sequencing analysis to identify differentially expressed lncRNAs, mRNAs, and miRNAs in the lesions and adjacent health tissues of patients with PTB. The results showed significant aberrant expression of lncRNAs, mRNAs, and miRNAs in the lesions. Patients with a history of TB likely exhibit an altered immune landscape compared to TB-naive individuals, which can arise from several factors, including but not limited to, the presence of immunological memory, and the alteration in lung architecture due to previous TB infections. Immunological memory, in particular, could lead to a heightened and more rapid immune response upon re-exposure to Mycobacterium tuberculosis, potentially altering the expression profiles of immune-related genes and pathways identified in our study.

Through miRNAs target prediction and correlation analysis, we identified a ceRNA regulatory network. Two networks were identified according to the differential expression of ceRNA transcripts. The first one consisted of LRRC34/hsa-miR-1185-1-3p/MAPK10 network, hsa-miR-1185-1-3p was up-regulated in PTB, and LRRC34 and MAPK10 were down regulated. In the second network consisting of P4HB/hsa-miR-549a-5p/LRBA, hsa-miR-549a-5p was found to be elevated in PTB and decreased in P4HB and LRBA. These data suggest that lncRNAs mediated ceRNA network, plays a key regulatory role in PTB. These results can enrich our understanding of the pathogenesis of PTB.

LRRC34 has been reported to play an important role in numerous cellular processes such as cell proliferation, differentiation, growth and cell survival^29^. SNPs at LRRC34 were found to promote interstitial lung abnormalities^30^. P4HB is more abundantly expressed in pulmonary artery muscle cells^31^. Previous studies have shown that overexpression of P4HB promotes liver cancer progression^32^, gastric cancer invasion and metastasis^33^, non-small cell lung cancer occurrence and growth^34^. MiR-1185-1-3p is significantly differentially expressed in bladder cancer^35^, Alzheimer’s disease^35^ and is involved in disease regulation. miR-549a-5p promotes tumorigenesis and metastasis by promoting angiogenesis through enhancing vascular permeability^36^. Until now, there have been no reports on LRRC34, P4HB, miR-1185-1-3p, and miR-549a-5p in PTB, although the results of our analysis argue for their significant involvement in the molecular regulation of PTB.

CD4 + T-cell deficiency and NK cell deficiency occur in lipopolysaccharide-responsive beige-like anchor (LRBA) protein deficiency, causing immunosuppression^37^. LRBA deficiency usually leads to recurrent infections, lymphoproliferative disorders, autoimmune diseases, allergic diseases^38^. Mitogen activated protein kinase 10 (MAPK10) is a member of the Jun terminal kinase subgroup of mitogen activated protein kinases^39^. The ceRNA network may be through inhibiting the expression of LRBA and MAPK10, causing immune function decline in patients, and thus promoting the occurrence of PTB. However, our results are inconsistent with those previously reported, Chen et al., found that MAPK10 is upregulated expression in tuberculosis^40^. This may also require an expanded sample size for validation.

Enrichment analysis showed that the differentially expressed mRNAs were involved in Th1 and Th2 cell differentiation, and Th17 cell differentiation. Interestingly, MAPK10 is also involved in these biological processes. Previous studies have shown that the percentage of Th1 cells is significantly reduced and the ratio of Th1/Th2 cells is imbalanced in patients with PTB, which leads to a decline in the body’s immune function^41^. Liu et al., also found a significant decrease in serum Th1 cytokine expression and a significant increase in Th2 cytokine expression in patients with PTB compared to controls^6^. These results suggest that the imbalance of Th1/Th2 cytokines is associated with PTB. Other studies have shown that active TB leads to the generation of mixed Th1/Th2^42^. Transcriptional and clinical analyses revealed that inhibition of Th17 responses is associated with the development of tuberculosis^43^. Low levels of interleukin-17 in serum have been associated with high mortality in patients with PTB^44^. However, it has also been shown that the Th17 response is enhanced in patients with TB, and Th17 is involved in the pathological process of TB^45^. This showed a significant imbalance in Th1, Th2, and Th17 cell differentiation in PTB, which may regulated by ceRNA networks, and contributes to the disease’s immune dysfunction.

This study also has certain limitations. First, the sample size of our study was small, and the follow-up needs to expand the sample size for in-depth exploration. Second, low-level expressed genes may have been missed by our analysis due to insufficient sequencing depth. Then, the potential heterogeneity in immune responses among our study population could contribute to variability in gene expression patterns, making it challenging to delineate the specific effects of current TB infection from the lingering impacts of past infections. Finally, although we performed molecular experiments to validate the significant profiling results, it was not enough to deeply delineate the ceRNA regulatory network’s mechanism of action.

## Conclusion

The lncRNAs associated ceRNA network was constructed by integrating lncRNAs, mRNAs, and miRNAs targeted interactions as well as their differential expression in the lesions and adjacent tissues of patients with PTB. They may contribute to PTB through regulatory effects of targeting Th1, Th2, and Th17 pathways for developing novel therapeutic strategies, offering new insights into the molecular mechanisms underlying PTB and paving the way for improved diagnostic and treatment approaches.

### Supplementary Information

Supplementary Figure S1.

Supplementary Table S1.

Supplementary Table S2.

## Data Availability

The datasets generated and analysed in the current study are available in the NCBI BioProject database (Accession PRJNA784123).

## Code availability

The codes during the current study are available from GitHub: https://github.com/GithubCDCode/TBceRNA_Python.
