# Supplementary figures and images for: Construction of ceRNA regulatory networks for active pulmonary tuberculosis
Source: Sci Rep. 2024 May 8;14:10595. doi: 10.1038/s41598-024-61451-2 (PMC11079045; doi:10.1038/s41598-024-61451-2)

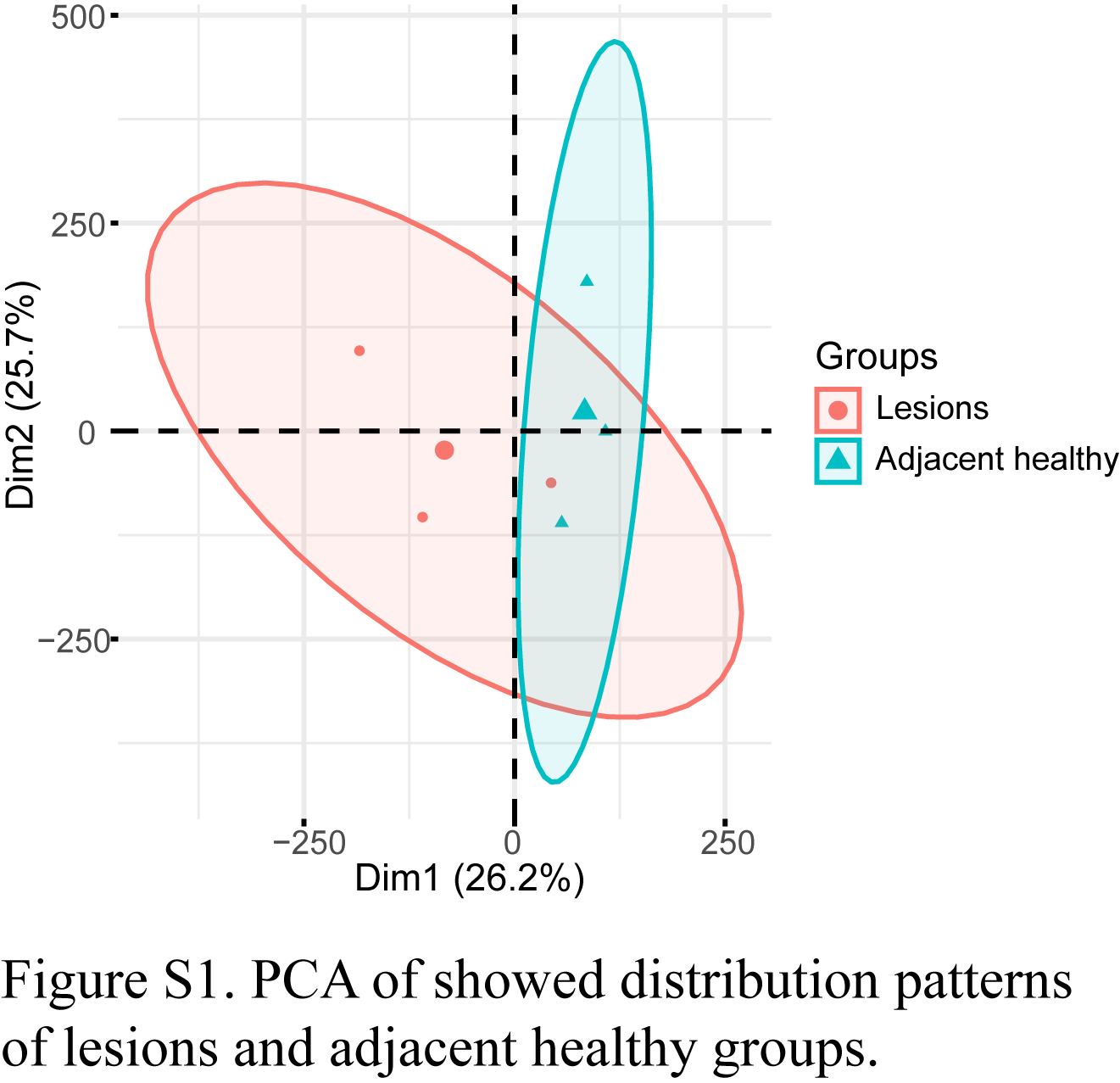

Supplement: Supplementary file 1 — Supplementary Figure S1. [file 41598_2024_61451_MOESM1_ESM.tif]
