# Supplementary material for: Construction of ceRNA regulatory networks for active pulmonary tuberculosis
Source: Sci Rep. 2024 May 8;14:10595. doi: 10.1038/s41598-024-61451-2 (PMC11079045; doi:10.1038/s41598-024-61451-2)
Supplement: Supplementary file 2 — Supplementary Table S1. [file 41598_2024_61451_MOESM2_ESM.docx]

Table S1. The primer sequences.

| Accession number | Genes | Primers |
| --- | --- | --- |
| KJ891221 | GAPDH | F: 5′-TGAAGGTCGGAGTCAACGGATTT-3′ |
|  |  | R: 5′-GCCATGGAATTTGCCATGGGTGG-3′ |
| NM_001172779 | LRRC34 | F: 5′-GGGCAGACTGTTGAAAAAGC-3′ |
|  |  | R: 5′-GTAACAGTACTGGGCCTGGAG-3′ |
| NM_000918 | P4HB | F: 5’-GGCTATCCCACCATCAAGTTC-3’ |
|  |  | R: 5’-TCACGATGTCATCAGCCTCTC-3’ |
| NG_013325 | MAPK10 | F: 5′-TTCTCAGGCACGGAATGG-3′ |
|  |  | R: 5′-TAAGTTGCCATAGTGAAGATCTGAG-3′ |
| NM_001367550 | LRBA | F: 5′-GAATGCACAGGAGGCAAATC-3′ |
|  |  | R: 5′-CCAGAAGCCACAGACGAGA-3′ |
| NR_004394 | U6 | F: 5′-GCTTCGGCAGCACATATACTAAAAT-3′ |
|  |  | R: 5′-CGCTTCACGAATTTGCGTGTCAT-3′ |
| LM383039 | miR-1185-1-3p | F: 5′-TTTGCTAGCCCGATGGATCACTTGGGCCT-3′ |
|  |  | R: 5′-TTATCTAGAGGAGGTACAGCCCCACTGTT-3′ |
| NR_030393 | miR-549a-5p | F: 5′-GATGAATTCCAGTAAATATCTTGTTTTTTCTA-3′ |
|  |  | R: 5′-GATGAATTCATATATTCCTAAAATAATGCTT-3′ |
